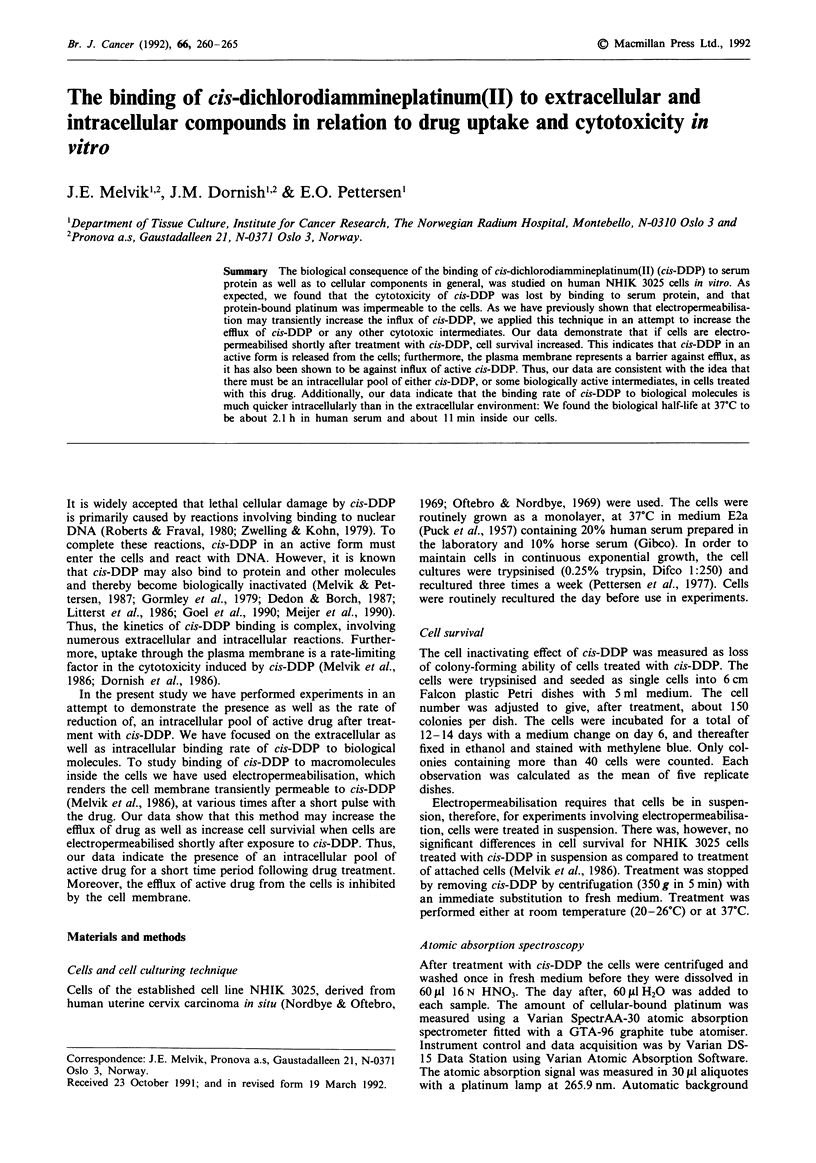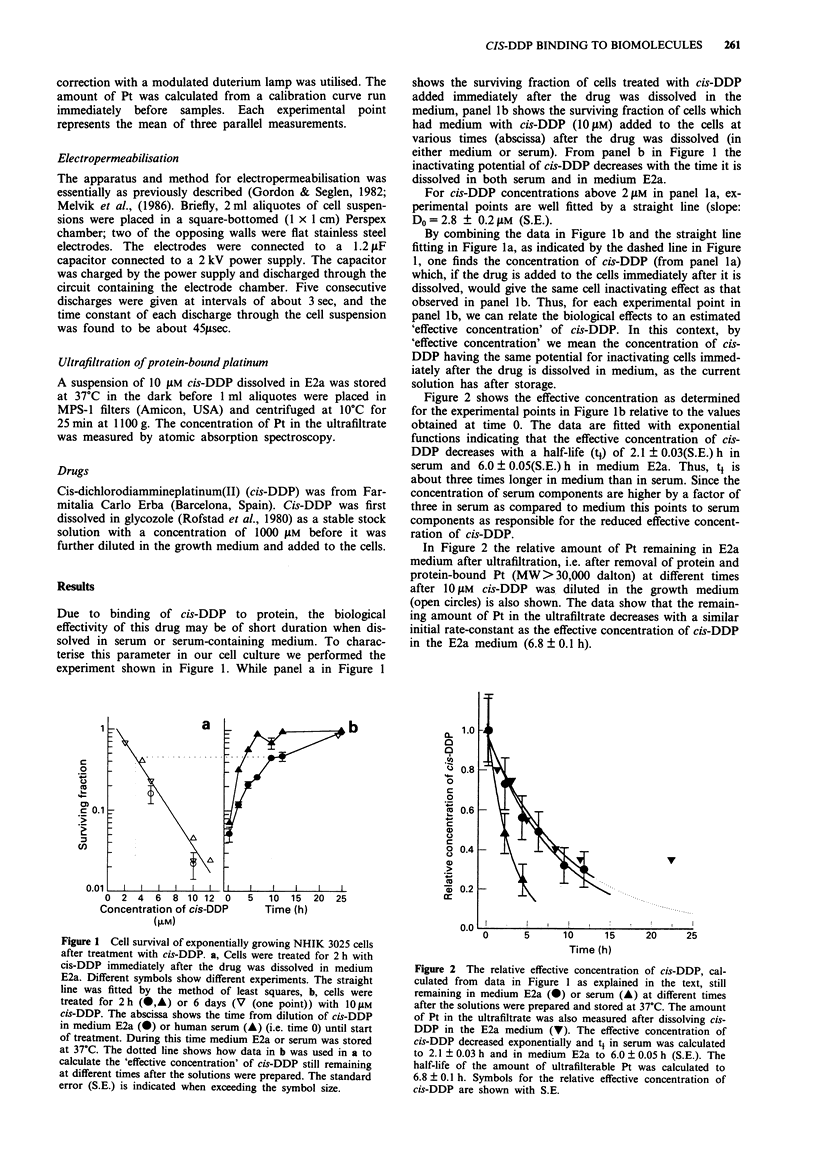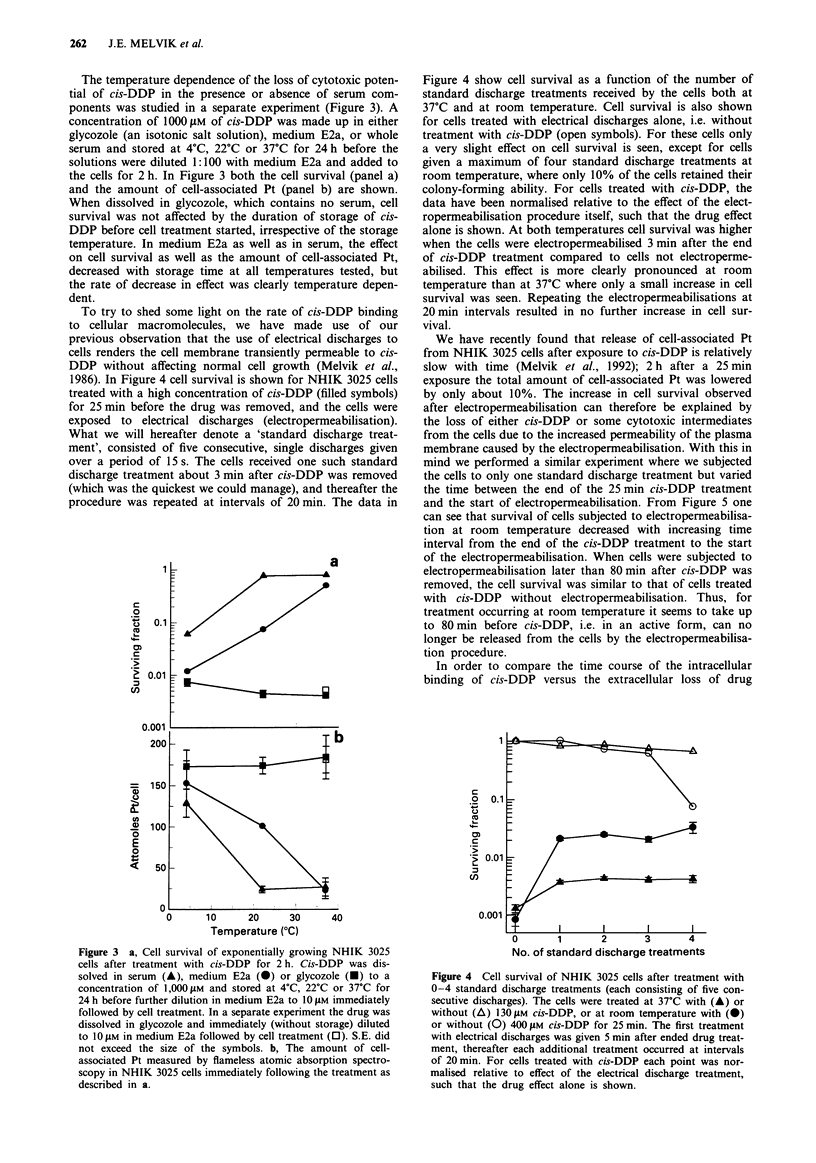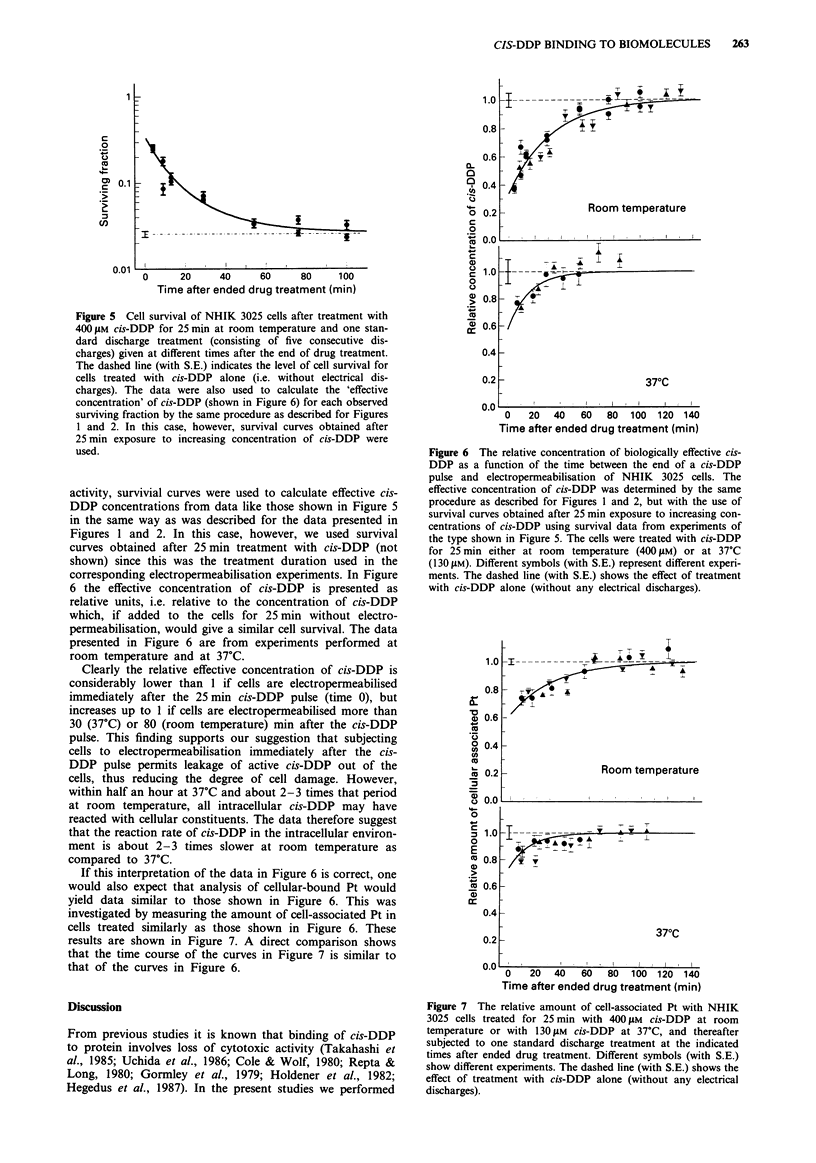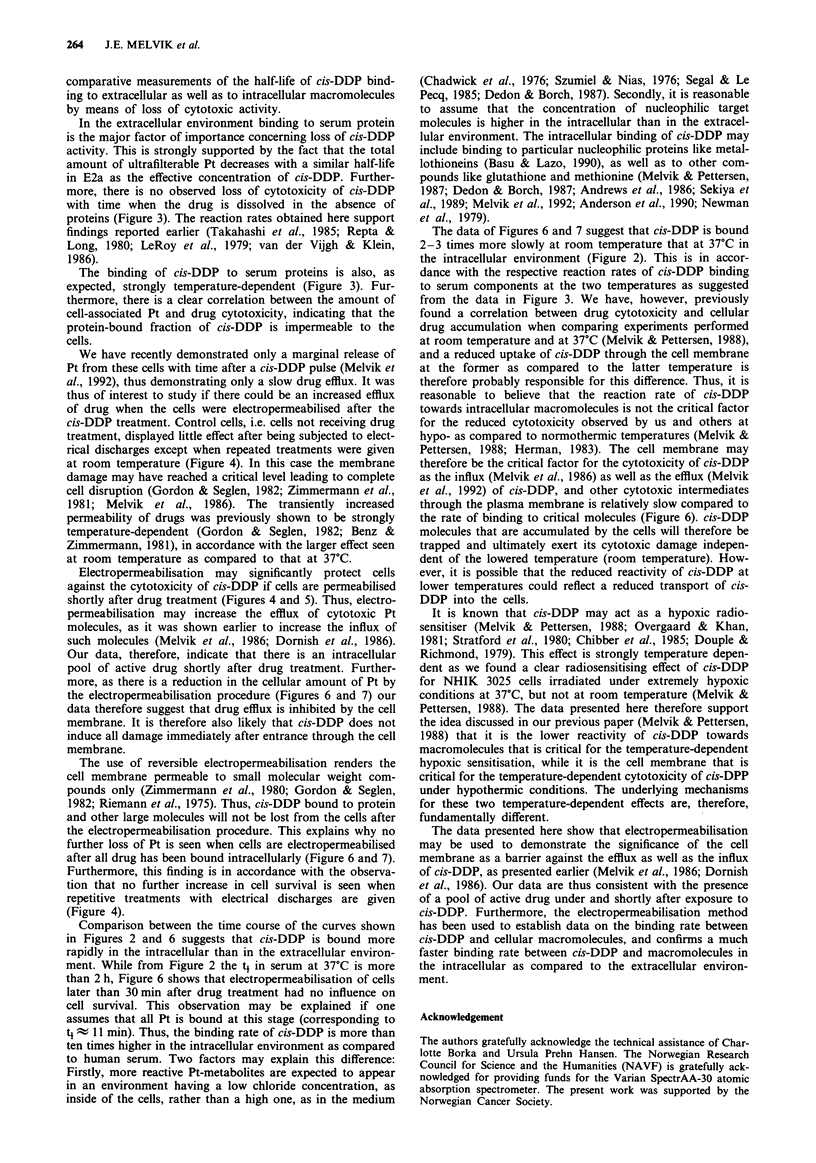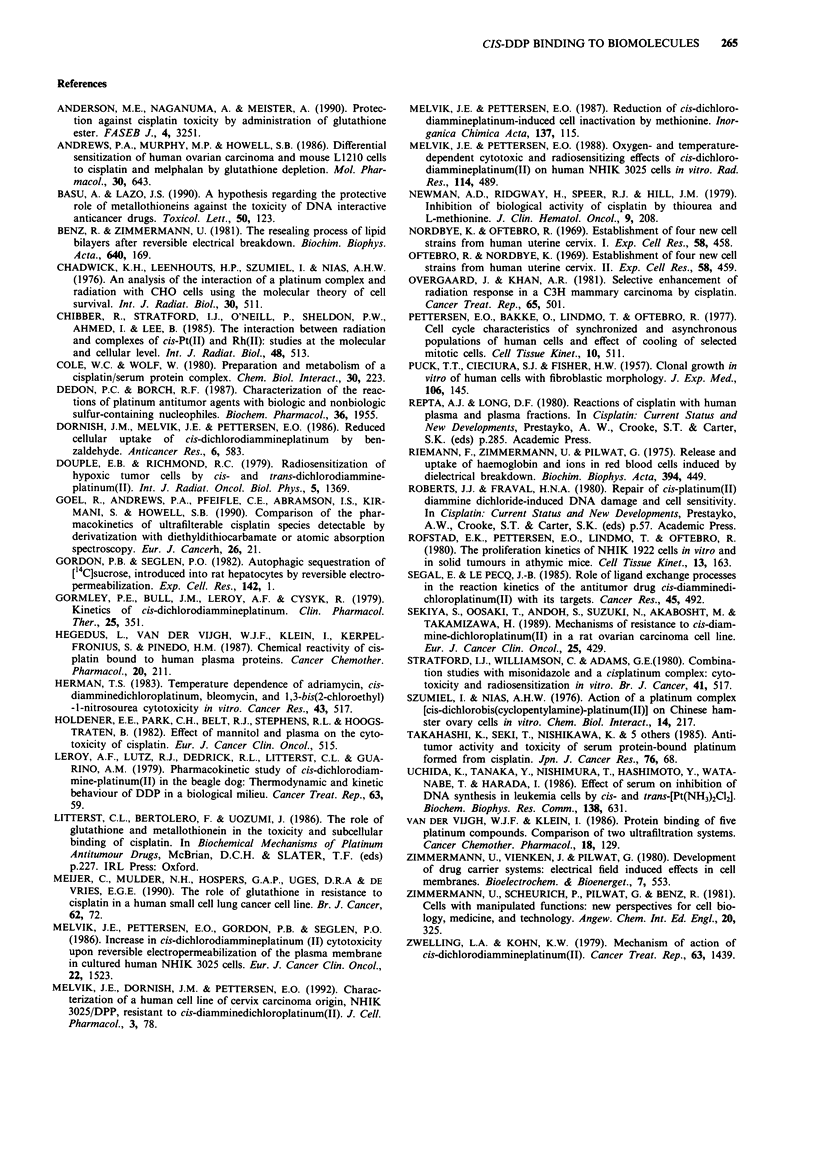# The binding of cis-dichlorodiammineplatinum(II) to extracellular and intracellular compounds in relation to drug uptake and cytotoxicity in vitro.

**DOI:** 10.1038/bjc.1992.254

**Published:** 1992-08

**Authors:** J. E. Melvik, J. M. Dornish, E. O. Pettersen

**Affiliations:** Department of Tissue Culture, Norwegian Radium Hospital, Montebello, Oslo.

## Abstract

The biological consequence of the binding of cis-dichlorodiammineplatinum(II) (cis-DDP) to serum protein as well as to cellular components in general, was studied on human NHIK 3025 cells in vitro. As expected, we found that the cytotoxicity of cis-DDP was lost by binding to serum protein, and that protein-bound platinum was impermeable to the cells. As we have previously shown that electropermeabilisation may transiently increase the influx of cis-DDP, we applied this technique in an attempt to increase the efflux of cis-DDP or any other cytotoxic intermediates. Our data demonstrate that if cells are electropermeabilised shortly after treatment with cis-DDP, cell survival increased. This indicates that cis-DDP in an active form is released from the cells; furthermore, the plasma membrane represents a barrier against efflux, as it has also been shown to be against influx of active cis-DDP. Thus, our data are consistent with the idea that there must be an intracellular pool of either cis-DDP, or some biologically active intermediates, in cells treated with this drug. Additionally, our data indicate that the binding rate of cis-DDP to biological molecules is much quicker intracellularly than in the extracellular environment: We found the biological half-life at 37 degrees C to be about 2.1 h in human serum and about 11 min inside our cells.


Br. J. Cancer (1992), 66, 260-265 © Macmillan Press Ltd., 1992

### The binding of *cis*-dichlorodiammineplatinum(II) to extracellular and intracellular compounds in relation to drug uptake and cytotoxicity *in vitro*

J.E. Melvik[1,2], J.M. Dornish[1,2] & E.O. Pettersen[1]

[1]*Department of Tissue Culture, Institute for Cancer Research, The Norwegian Radium Hospital, Montebello, N-0310 Oslo 3 and* [2]*Pronova a.s, Gaustadalleen 21, N-0371 Oslo 3, Norway.*

**Summary** The biological consequence of the binding of *cis*-dichlorodiammineplatinum(II) (*cis*-DDP) to serum protein as well as to cellular components in general, was studied on human NHIK 3025 cells *in vitro*. As expected, we found that the cytotoxicity of *cis*-DDP was lost by binding to serum protein, and that protein-bound platinum was impermeable to the cells. As we have previously shown that electropermeabilisation may transiently increase the influx of *cis*-DDP, we applied this technique in an attempt to increase the efflux of *cis*-DDP or any other cytotoxic intermediates. Our data demonstrate that if cells are electropermeabilised shortly after treatment with *cis*-DDP, cell survival increased. This indicates that *cis*-DDP in an active form is released from the cells; furthermore, the plasma membrane represents a barrier against efflux, as it has also been shown to be against influx of active *cis*-DDP. Thus, our data are consistent with the idea that there must be an intracellular pool of either *cis*-DDP, or some biologically active intermediates, in cells treated with this drug. Additionally, our data indicate that the binding rate of *cis*-DDP to biological molecules is much quicker intracellularly than in the extracellular environment: We found the biological half-life at 37°C to be about 2.1 h in human serum and about 11 min inside our cells.

It is widely accepted that lethal cellular damage by *cis*-DDP is primarily caused by reactions involving binding to nuclear DNA (Roberts & Fraval, 1980; Zwelling & Kohn, 1979). To complete these reactions, *cis*-DDP in an active form must enter the cells and react with DNA. However, it is known that *cis*-DDP may also bind to protein and other molecules and thereby become biologically inactivated (Melvik & Pettersen, 1987; Gormley *et al.*, 1979; Dedon & Borch, 1987; Litterst *et al.*, 1986; Goel *et al.*, 1990; Meijer *et al.*, 1990). Thus, the kinetics of *cis*-DDP binding is complex, involving numerous extracellular and intracellular reactions. Furthermore, uptake through the plasma membrane is a rate-limiting factor in the cytotoxicity induced by *cis*-DDP (Melvik *et al.*, 1986; Dornish *et al.*, 1986).

In the present study we have performed experiments in an attempt to demonstrate the presence as well as the rate of reduction of, an intracellular pool of active drug after treatment with *cis*-DDP. We have focused on the extracellular as well as intracellular binding rate of *cis*-DDP to biological molecules. To study binding of *cis*-DDP to macromolecules inside the cells we have used electropermeabilisation, which renders the cell membrane transiently permeable to *cis*-DDP (Melvik *et al.*, 1986), at various times after a short pulse with the drug. Our data show that this method may increase the efflux of drug as well as increase cell survivial when cells are electropermeabilised shortly after exposure to *cis*-DDP. Thus, our data indicate the presence of an intracellular pool of active drug for a short time period following drug treatment. Moreover, the efflux of active drug from the cells is inhibited by the cell membrane.

### Materials and methods

### Cells and cell culturing technique

Cells of the established cell line NHIK 3025, derived from human uterine cervix carcinoma *in situ* (Nordbye & Oftebro, 1969; Oftebro & Nordbye, 1969) were used. The cells were routinely grown as a monolayer, at 37°C in medium E2a (Puck *et al.*, 1957) containing 20% human serum prepared in the laboratory and 10% horse serum (Gibco). In order to maintain cells in continuous exponential growth, the cell cultures were trypsinised (0.25% trypsin, Difco 1:250) and recultured three times a week (Pettersen *et al.*, 1977). Cells were routinely recultured the day before use in experiments.

### Cell survival

The cell inactivating effect of *cis*-DDP was measured as loss of colony-forming ability of cells treated with *cis*-DDP. The cells were trypsinised and seeded as single cells into 6 cm Falcon plastic Petri dishes with 5 ml medium. The cell number was adjusted to give, after treatment, about 150 colonies per dish. The cells were incubated for a total of 12-14 days with a medium change on day 6, and thereafter fixed in ethanol and stained with methylene blue. Only colonies containing more than 40 cells were counted. Each observation was calculated as the mean of five replicate dishes.

Electropermeabilisation requires that cells be in suspension, therefore, for experiments involving electropermeabilisation, cells were treated in suspension. There was, however, no significant differences in cell survival for NHIK 3025 cells treated with *cis*-DDP in suspension as compared to treatment of attached cells (Melvik *et al.*, 1986). Treatment was stopped by removing *cis*-DDP by centrifugation (350 *g* in 5 min) with an immediate substitution to fresh medium. Treatment was performed either at room temperature (20-26°C) or at 37°C.

### Atomic absorption spectroscopy

After treatment with *cis*-DDP the cells were centrifuged and washed once in fresh medium before they were dissolved in 60 µl 16 N $HNO_3$. The day after, 60 µl $H_2O$ was added to each sample. The amount of cellular-bound platinum was measured using a Varian SpectrAA-30 atomic absorption spectrometer fitted with a GTA-96 graphite tube atomiser. Instrument control and data acquisition was by Varian DS-15 Data Station using Varian Atomic Absorption Software. The atomic absorption signal was measured in 30 µl aliquotes with a platinum lamp at 265.9 nm. Automatic background

Correspondence: J.E. Melvik, Pronova a.s, Gaustadalleen 21, N-0371 Oslo 3, Norway.
Received 23 October 1991; and in revised form 19 March 1992.

correction with a modulated duterium lamp was utilised. The amount of Pt was calculated from a calibration curve run immediately before samples. Each experimental point represents the mean of three parallel measurements.

*Electropermeabilisation*

The apparatus and method for electropermeabilisation was essentially as previously described (Gordon & Seglen, 1982; Melvik *et al.*, (1986). Briefly, 2 ml aliquotes of cell suspensions were placed in a square-bottomed (1 × 1 cm) Perspex chamber; two of the opposing walls were flat stainless steel electrodes. The electrodes were connected to a 1.2 μF capacitor connected to a 2 kV power supply. The capacitor was charged by the power supply and discharged through the circuit containing the electrode chamber. Five consecutive discharges were given at intervals of about 3 sec, and the time constant of each discharge through the cell suspension was found to be about 45μsec.

*Ultrafiltration of protein-bound platinum*

A suspension of 10 μM *cis*-DDP dissolved in E2a was stored at 37°C in the dark before 1 ml aliquotes were placed in MPS-1 filters (Amicon, USA) and centrifuged at 10°C for 25 min at 1100 g. The concentration of Pt in the ultrafiltrate was measured by atomic absorption spectroscopy.

*Drugs*

Cis-dichlorodiammineplatinum(II) (*cis*-DDP) was from Farmitalia Carlo Erba (Barcelona, Spain). *Cis*-DDP was first dissolved in glycozole (Rofstad *et al.*, 1980) as a stable stock solution with a concentration of 1000 μM before it was further diluted in the growth medium and added to the cells.

**Results**

Due to binding of *cis*-DDP to protein, the biological effectivity of this drug may be of short duration when dissolved in serum or serum-containing medium. To characterise this parameter in our cell culture we performed the experiment shown in Figure 1. While panel a in Figure 1 shows the surviving fraction of cells treated with *cis*-DDP added immediately after the drug was dissolved in the medium, panel 1b shows the surviving fraction of cells which had medium with *cis*-DDP (10 μM) added to the cells at various times (abscissa) after the drug was dissolved (in either medium or serum). From panel b in Figure 1 the inactivating potential of *cis*-DDP decreases with the time it is dissolved in both serum and in medium E2a.

For *cis*-DDP concentrations above 2 μM in panel 1a, experimental points are well fitted by a straight line (slope: $D_0 = 2.8 \pm 0.2$ μM (S.E.).

By combining the data in Figure 1b and the straight line fitting in Figure 1a, as indicated by the dashed line in Figure 1, one finds the concentration of *cis*-DDP (from panel 1a) which, if the drug is added to the cells immediately after it is dissolved, would give the same cell inactivating effect as that observed in panel 1b. Thus, for each experimental point in panel 1b, we can relate the biological effects to an estimated 'effective concentration' of *cis*-DDP. In this context, by 'effective concentration' we mean the concentration of *cis*-DDP having the same potential for inactivating cells immediately after the drug is dissolved in medium, as the current solution has after storage.

Figure 2 shows the effective concentration as determined for the experimental points in Figure 1b relative to the values obtained at time 0. The data are fitted with exponential functions indicating that the effective concentration of *cis*-DDP decreases with a half-life ($t_{½}$) of 2.1 ± 0.03(S.E.) h in serum and 6.0 ± 0.05(S.E.) h in medium E2a. Thus, $t_{½}$ is about three times longer in medium than in serum. Since the concentration of serum components are higher by a factor of three in serum as compared to medium this points to serum components as responsible for the reduced effective concentration of *cis*-DDP.

In Figure 2 the relative amount of Pt remaining in E2a medium after ultrafiltration, i.e. after removal of protein and protein-bound Pt (MW > 30,000 dalton) at different times after 10 μM *cis*-DDP was diluted in the growth medium (open circles) is also shown. The data show that the remaining amount of Pt in the ultrafiltrate decreases with a similar initial rate-constant as the effective concentration of *cis*-DDP in the E2a medium (6.8 ± 0.1 h).

**Figure 1** Cell survival of exponentially growing NHIK 3025 cells after treatment with *cis*-DDP. **a**, Cells were treated for 2 h with cis-DDP immediately after the drug was dissolved in medium E2a. Different symbols show different experiments. The straight line was fitted by the method of least squares, **b**, cells were treated for 2 h (●,▲) or 6 days (∇ (one point)) with 10 μM *cis*-DDP. The abscissa shows the time from dilution of *cis*-DDP in medium E2a (●) or human serum (▲) (i.e. time 0) until start of treatment. During this time medium E2a or serum was stored at 37°C. The dotted line shows how data in **b** was used in **a** to calculate the 'effective concentration' of *cis*-DDP still remaining at different times after the solutions were prepared. The standard error (S.E.) is indicated when exceeding the symbol size.

**Figure 2** The relative effective concentration of *cis*-DDP, calculated from data in Figure 1 as explained in the text, still remaining in medium E2a (●) or serum (▲) at different times after the solutions were prepared and stored at 37°C. The amount of Pt in the ultrafiltrate was also measured after dissolving *cis*-DDP in the E2a medium (▼). The effective concentration of *cis*-DDP decreased exponentially and $t_{½}$ in serum was calculated to 2.1 ± 0.03 h and in medium E2a to 6.0 ± 0.05 h (S.E.). The half-life of the amount of ultrafilterable Pt was calculated to 6.8 ± 0.1 h. Symbols for the relative effective concentration of *cis*-DDP are shown with S.E.

The temperature dependence of the loss of cytotoxic potential of *cis*-DDP in the presence or absence of serum components was studied in a separate experiment (Figure 3). A concentration of 1000 µM of *cis*-DDP was made up in either glycozole (an isotonic salt solution), medium E2a, or whole serum and stored at 4°C, 22°C or 37°C for 24 h before the solutions were diluted 1:100 with medium E2a and added to the cells for 2 h. In Figure 3 both the cell survival (panel a) and the amount of cell-associated Pt (panel b) are shown. When dissolved in glycozole, which contains no serum, cell survival was not affected by the duration of storage of *cis*-DDP before cell treatment started, irrespective of the storage temperature. In medium E2a as well as in serum, the effect on cell survival as well as the amount of cell-associated Pt, decreased with storage time at all temperatures tested, but the rate of decrease in effect was clearly temperature dependent.

To try to shed some light on the rate of *cis*-DDP binding to cellular macromolecules, we have made use of our previous observation that the use of electrical discharges to cells renders the cell membrane transiently permeable to *cis*-DDP without affecting normal cell growth (Melvik *et al.*, 1986). In Figure 4 cell survival is shown for NHIK 3025 cells treated with a high concentration of *cis*-DDP (filled symbols) for 25 min before the drug was removed, and the cells were exposed to electrical discharges (electropermeabilisation). What we will hereafter denote a 'standard discharge treatment', consisted of five consecutive, single discharges given over a period of 15 s. The cells received one such standard discharge treatment about 3 min after *cis*-DDP was removed (which was the quickest we could manage), and thereafter the procedure was repeated at intervals of 20 min. The data in Figure 4 show cell survival as a function of the number of standard discharge treatments received by the cells both at 37°C and at room temperature. Cell survival is also shown for cells treated with electrical discharges alone, i.e. without treatment with *cis*-DDP (open symbols). For these cells only a very slight effect on cell survival is seen, except for cells given a maximum of four standard discharge treatments at room temperature, where only 10% of the cells retained their colony-forming ability. For cells treated with *cis*-DDP, the data have been normalised relative to the effect of the electropermeabilisation procedure itself, such that the drug effect alone is shown. At both temperatures cell survival was higher when the cells were electropermeabilised 3 min after the end of *cis*-DDP treatment compared to cells not electropermeabilised. This effect is more clearly pronounced at room temperature than at 37°C where only a small increase in cell survival was seen. Repeating the electropermeabilisations at 20 min intervals resulted in no further increase in cell survival.

We have recently found that release of cell-associated Pt from NHIK 3025 cells after exposure to *cis*-DDP is relatively slow with time (Melvik *et al.*, 1992); 2 h after a 25 min exposure the total amount of cell-associated Pt was lowered by only about 10%. The increase in cell survival observed after electropermeabilisation can therefore be explained by the loss of either *cis*-DDP or some cytotoxic intermediates from the cells due to the increased permeability of the plasma membrane caused by the electropermeabilisation. With this in mind we performed a similar experiment where we subjected the cells to only one standard discharge treatment but varied the time between the end of the 25 min *cis*-DDP treatment and the start of electropermeabilisation. From Figure 5 one can see that survival of cells subjected to electropermeabilisation at room temperature decreased with increasing time interval from the end of the *cis*-DDP treatment to the start of the electropermeabilisation. When cells were subjected to electropermeabilisation later than 80 min after *cis*-DDP was removed, the cell survival was similar to that of cells treated with *cis*-DDP without electropermeabilisation. Thus, for treatment occurring at room temperature it seems to take up to 80 min before *cis*-DDP, i.e. in an active form, can no longer be released from the cells by the electropermeabilisation procedure.

In order to compare the time course of the intracellular binding of *cis*-DDP versus the extracellular loss of drug

**Figure 3** **a**, Cell survival of exponentially growing NHIK 3025 cells after treatment with *cis*-DDP for 2 h. *Cis*-DDP was dissolved in serum (▲), medium E2a (●) or glycozole (■) to a concentration of 1,000 µM and stored at 4°C, 22°C or 37°C for 24 h before further dilution in medium E2a to 10 µM immediately followed by cell treatment. In a separate experiment the drug was dissolved in glycozole and immediately (without storage) diluted to 10 µM in medium E2a followed by cell treatment (□). S.E. did not exceed the size of the symbols. **b**, The amount of cell-associated Pt measured by flameless atomic absorption spectroscopy in NHIK 3025 cells immediately following the treatment as described in **a**.

**Figure 4** Cell survival of NHIK 3025 cells after treatment with 0–4 standard discharge treatments (each consisting of five consecutive discharges). The cells were treated at 37°C with (▲) or without (△) 130 µM *cis*-DDP, or at room temperature with (●) or without (○) 400 µM *cis*-DDP for 25 min. The first treatment with electrical discharges was given 5 min after ended drug treatment, thereafter each additional treatment occurred at intervals of 20 min. For cells treated with *cis*-DDP each point was normalised relative to effect of the electrical discharge treatment, such that the drug effect alone is shown.

**Figure 5** Cell survival of NHIK 3025 cells after treatment with 400 μM *cis*-DDP for 25 min at room temperature and one standard discharge treatment (consisting of five consecutive discharges) given at different times after the end of drug treatment. The dashed line (with S.E.) indicates the level of cell survival for cells treated with *cis*-DDP alone (i.e. without electrical discharges). The data were also used to calculate the 'effective concentration' of *cis*-DDP (shown in Figure 6) for each observed surviving fraction by the same procedure as described for Figures 1 and 2. In this case, however, survival curves obtained after 25 min exposure to increasing concentration of *cis*-DDP were used.

**Figure 6** The relative concentration of biologically effective *cis*-DDP as a function of the time between the end of a *cis*-DDP pulse and electropermeabilisation of NHIK 3025 cells. The effective concentration of *cis*-DDP was determined by the same procedure as described for Figures 1 and 2, but with the use of survival curves obtained after 25 min exposure to increasing concentrations of *cis*-DDP using survival data from experiments of the type shown in Figure 5. The cells were treated with *cis*-DDP for 25 min either at room temperature (400 μM) or at 37°C (130 μM). Different symbols (with S.E.) represent different experiments. The dashed line (with S.E.) shows the effect of treatment with *cis*-DDP alone (without any electrical discharges).

activity, survivial curves were used to calculate effective *cis*-DDP concentrations from data like those shown in Figure 5 in the same way as was described for the data presented in Figures 1 and 2. In this case, however, we used survival curves obtained after 25 min treatment with *cis*-DDP (not shown) since this was the treatment duration used in the corresponding electropermeabilisation experiments. In Figure 6 the effective concentration of *cis*-DDP is presented as relative units, i.e. relative to the concentration of *cis*-DDP which, if added to the cells for 25 min without electropermeabilisation, would give a similar cell survival. The data presented in Figure 6 are from experiments performed at room temperature and at 37°C.

Clearly the relative effective concentration of *cis*-DDP is considerably lower than 1 if cells are electropermeabilised immediately after the 25 min *cis*-DDP pulse (time 0), but increases up to 1 if cells are electropermeabilised more than 30 (37°C) or 80 (room temperature) min after the *cis*-DDP pulse. This finding supports our suggestion that subjecting cells to electropermeabilisation immediately after the *cis*-DDP pulse permits leakage of active *cis*-DDP out of the cells, thus reducing the degree of cell damage. However, within half an hour at 37°C and about 2–3 times that period at room temperature, all intracellular *cis*-DDP may have reacted with cellular constituents. The data therefore suggest that the reaction rate of *cis*-DDP in the intracellular environment is about 2–3 times slower at room temperature as compared to 37°C.

If this interpretation of the data in Figure 6 is correct, one would also expect that analysis of cellular-bound Pt would yield data similar to those shown in Figure 6. This was investigated by measuring the amount of cell-associated Pt in cells treated similarly as those shown in Figure 6. These results are shown in Figure 7. A direct comparison shows that the time course of the curves in Figure 7 is similar to that of the curves in Figure 6.

**Figure 7** The relative amount of cell-associated Pt with NHIK 3025 cells treated for 25 min with 400 μM *cis*-DDP at room temperature or with 130 μM *cis*-DDP at 37°C, and thereafter subjected to one standard discharge treatment at the indicated times after ended drug treatment. Different symbols (with S.E.) show different experiments. The dashed line (with S.E.) shows the effect of treatment with *cis*-DDP alone (without any electrical discharges).

### Discussion

From previous studies it is known that binding of *cis*-DDP to protein involves loss of cytotoxic activity (Takahashi *et al.*, 1985; Uchida *et al.*, 1986; Cole & Wolf, 1980; Repta & Long, 1980; Gormley *et al.*, 1979; Holdener *et al.*, 1982; Hegedus *et al.*, 1987). In the present studies we performed comparative measurements of the half-life of *cis*-DDP binding to extracellular as well as to intracellular macromolecules by means of loss of cytotoxic activity.

In the extracellular environment binding to serum protein is the major factor of importance concerning loss of *cis*-DDP activity. This is strongly supported by the fact that the total amount of ultrafilterable Pt decreases with a similar half-life in E2a as the effective concentration of *cis*-DDP. Furthermore, there is no observed loss of cytotoxicity of *cis*-DDP with time when the drug is dissolved in the absence of proteins (Figure 3). The reaction rates obtained here support findings reported earlier (Takahashi *et al.*, 1985; Repta & Long, 1980; LeRoy *et al.*, 1979; van der Vijgh & Klein, 1986).

The binding of *cis*-DDP to serum proteins is also, as expected, strongly temperature-dependent (Figure 3). Furthermore, there is a clear correlation between the amount of cell-associated Pt and drug cytotoxicity, indicating that the protein-bound fraction of *cis*-DDP is impermeable to the cells.

We have recently demonstrated only a marginal release of Pt from these cells with time after a *cis*-DDP pulse (Melvik *et al.*, 1992), thus demonstrating only a slow drug efflux. It was thus of interest to study if there could be an increased efflux of drug when the cells were electropermeabilised after the *cis*-DDP treatment. Control cells, i.e. cells not receiving drug treatment, displayed little effect after being subjected to electrical discharges except when repeated treatments were given at room temperature (Figure 4). In this case the membrane damage may have reached a critical level leading to complete cell disruption (Gordon & Seglen, 1982; Zimmermann *et al.*, 1981; Melvik *et al.*, 1986). The transiently increased permeability of drugs was previously shown to be strongly temperature-dependent (Gordon & Seglen, 1982; Benz & Zimmermann, 1981), in accordance with the larger effect seen at room temperature as compared to that at 37°C.

Electropermeabilisation may significantly protect cells against the cytotoxicity of *cis*-DDP if cells are permeabilised shortly after drug treatment (Figures 4 and 5). Thus, electropermeabilisation may increase the efflux of cytotoxic Pt molecules, as it was shown earlier to increase the influx of such molecules (Melvik *et al.*, 1986; Dornish *et al.*, 1986). Our data, therefore, indicate that there is an intracellular pool of active drug shortly after drug treatment. Furthermore, as there is a reduction in the cellular amount of Pt by the electropermeabilisation procedure (Figures 6 and 7) our data therefore suggest that drug efflux is inhibited by the cell membrane. It is therefore also likely that *cis*-DDP does not induce all damage immediately after entrance through the cell membrane.

The use of reversible electropermeabilisation renders the cell membrane permeable to small molecular weight compounds only (Zimmermann *et al.*, 1980; Gordon & Seglen, 1982; Riemann *et al.*, 1975). Thus, *cis*-DDP bound to protein and other large molecules will not be lost from the cells after the electropermeabilisation procedure. This explains why no further loss of Pt is seen when cells are electropermeabilised after all drug has been bound intracellularly (Figure 6 and 7). Furthermore, this finding is in accordance with the observation that no further increase in cell survival is seen when repetitive treatments with electrical discharges are given (Figure 4).

Comparison between the time course of the curves shown in Figures 2 and 6 suggests that *cis*-DDP is bound more rapidly in the intracellular than in the extracellular environment. While from Figure 2 the $t_{1/2}$ in serum at 37°C is more than 2 h, Figure 6 shows that electropermeabilisation of cells later than 30 min after drug treatment had no influence on cell survival. This observation may be explained if one assumes that all Pt is bound at this stage (corresponding to $t_{1/2} \approx 11$ min). Thus, the binding rate of *cis*-DDP is more than ten times higher in the intracellular environment as compared to human serum. Two factors may explain this difference: Firstly, more reactive Pt-metabolites are expected to appear in an environment having a low chloride concentration, as inside of the cells, rather than a high one, as in the medium (Chadwick *et al.*, 1976; Szumiel & Nias, 1976; Segal & Le Pecq, 1985; Dedon & Borch, 1987). Secondly, it is reasonable to assume that the concentration of nucleophilic target molecules is higher in the intracellular than in the extracellular environment. The intracellular binding of *cis*-DDP may include binding to particular nucleophilic proteins like metallothioneins (Basu & Lazo, 1990), as well as to other compounds like glutathione and methionine (Melvik & Pettersen, 1987; Dedon & Borch, 1987; Andrews *et al.*, 1986; Sekiya *et al.*, 1989; Melvik *et al.*, 1992; Anderson *et al.*, 1990; Newman *et al.*, 1979).

The data of Figures 6 and 7 suggest that *cis*-DDP is bound 2–3 times more slowly at room temperature that at 37°C in the intracellular environment (Figure 2). This is in accordance with the respective reaction rates of *cis*-DDP binding to serum components at the two temperatures as suggested from the data in Figure 3. We have, however, previously found a correlation between drug cytotoxicity and cellular drug accumulation when comparing experiments performed at room temperature and at 37°C (Melvik & Pettersen, 1988), and a reduced uptake of *cis*-DDP through the cell membrane at the former as compared to the latter temperature is therefore probably responsible for this difference. Thus, it is reasonable to believe that the reaction rate of *cis*-DDP towards intracellular macromolecules is not the critical factor for the reduced cytotoxicity observed by us and others at hypo- as compared to normothermic temperatures (Melvik & Pettersen, 1988; Herman, 1983). The cell membrane may therefore be the critical factor for the cytotoxicity of *cis*-DDP as the influx (Melvik *et al.*, 1986) as well as the efflux (Melvik *et al.*, 1992) of *cis*-DDP, and other cytotoxic intermediates through the plasma membrane is relatively slow compared to the rate of binding to critical molecules (Figure 6). *cis*-DDP molecules that are accumulated by the cells will therefore be trapped and ultimately exert its cytotoxic damage independent of the lowered temperature (room temperature). However, it is possible that the reduced reactivity of *cis*-DDP at lower temperatures could reflect a reduced transport of *cis*-DDP into the cells.

It is known that *cis*-DDP may act as a hypoxic radiosensitiser (Melvik & Pettersen, 1988; Overgaard & Khan, 1981; Stratford *et al.*, 1980; Chibber *et al.*, 1985; Douple & Richmond, 1979). This effect is strongly temperature dependent as we found a clear radiosensitising effect of *cis*-DDP for NHIK 3025 cells irradiated under extremely hypoxic conditions at 37°C, but not at room temperature (Melvik & Pettersen, 1988). The data presented here therefore support the idea discussed in our previous paper (Melvik & Pettersen, 1988) that it is the lower reactivity of *cis*-DDP towards macromolecules that is critical for the temperature-dependent hypoxic sensitisation, while it is the cell membrane that is critical for the temperature-dependent cytotoxicity of *cis*-DPP under hypothermic conditions. The underlying mechanisms for these two temperature-dependent effects are, therefore, fundamentally different.

The data presented here show that electropermeabilisation may be used to demonstrate the significance of the cell membrane as a barrier against the efflux as well as the influx of *cis*-DDP, as presented earlier (Melvik *et al.*, 1986; Dornish *et al.*, 1986). Our data are thus consistent with the presence of a pool of active drug under and shortly after exposure to *cis*-DDP. Furthermore, the electropermeabilisation method has been used to establish data on the binding rate between *cis*-DDP and cellular macromolecules, and confirms a much faster binding rate between *cis*-DDP and macromolecules in the intracellular as compared to the extracellular environment.

**Acknowledgement**

The authors gratefully acknowledge the technical assistance of Charlotte Borka and Ursula Prehn Hansen. The Norwegian Research Council for Science and the Humanities (NAVF) is gratefully acknowledged for providing funds for the Varian SpectrAA-30 atomic absorption spectrometer. The present work was supported by the Norwegian Cancer Society.

### References

ANDERSON, M.E., NAGANUMA, A. & MEISTER, A. (1990). Protection against cisplatin toxicity by administration of glutathione ester. *FASEB J.*, **4**, 3251.

ANDREWS, P.A., MURPHY, M.P. & HOWELL, S.B. (1986). Differential sensitization of human ovarian carcinoma and mouse L1210 cells to cisplatin and melphalan by glutathione depletion. *Mol. Pharmacol.*, **30**, 643.

BASU, A. & LAZO, J.S. (1990). A hypothesis regarding the protective role of metallothioneins against the toxicity of DNA interactive anticancer drugs. *Toxicol. Lett.*, **50**, 123.

BENZ, R. & ZIMMERMANN, U. (1981). The resealing process of lipid bilayers after reversible electrical breakdown. *Biochim. Biophys. Acta.*, **640**, 169.

CHADWICK, K.H., LEENHOUTS, H.P., SZUMIEL, I. & NIAS, A.H.W. (1976). An analysis of the interaction of a platinum complex and radiation with CHO cells using the molecular theory of cell survival. *Int. J. Radiat. Biol.*, **30**, 511.

CHIBBER, R., STRATFORD, I.J., O'NEILL, P., SHELDON, P.W., AHMED, I. & LEE, B. (1985). The interaction between radiation and complexes of *cis*-Pt(II) and Rh(II): studies at the molecular and cellular level. *Int. J. Radiat. Biol.*, **48**, 513.

COLE, W.C. & WOLF, W. (1980). Preparation and metabolism of a cisplatin/serum protein complex. *Chem. Biol. Interact.*, **30**, 223.

DEDON, P.C. & BORCH, R.F. (1987). Characterization of the reactions of platinum antitumor agents with biologic and nonbiologic sulfur-containing nucleophiles. *Biochem. Pharmacol.*, **36**, 1955.

DORNISH, J.M., MELVIK, J.E. & PETTERSEN, E.O. (1986). Reduced cellular uptake of *cis*-dichlorodiammineplatinum by benzaldehyde. *Anticancer Res.*, **6**, 583.

DOUPLE, E.B. & RICHMOND, R.C. (1979). Radiosensitization of hypoxic tumor cells by *cis*- and *trans*-dichlorodiammine-platinum(II). *Int. J. Radiat. Oncol. Biol. Phys.*, **5**, 1369.

GOEL, R., ANDREWS, P.A., PFEIFLE, C.E., ABRAMSON, I.S., KIRMANI, S. & HOWELL, S.B. (1990). Comparison of the pharmacokinetics of ultrafilterable cisplatin species detectable by derivatization with diethyldithiocarbamate or atomic absorption spectroscopy. *Eur. J. Cancerh*, **26**, 21.

GORDON, P.B. & SEGLEN, P.O. (1982). Autophagic sequestration of [$^{14}$C]sucrose, introduced into rat hepatocytes by reversible electropermeabilization. *Exp. Cell. Res.*, **142**, 1.

GORMLEY, P.E., BULL, J.M., LEROY, A.F. & CYSYK, R. (1979). Kinetics of *cis*-dichlorodiammineplatinum. *Clin. Pharmacol. Ther.*, **25**, 351.

HEGEDUS, L., VAN DER VIJGH, W.J.F., KLEIN, I., KERPEL-FRONIUS, S. & PINEDO, H.M. (1987). Chemical reactivity of cisplatin bound to human plasma proteins. *Cancer Chemother. Pharmacol.*, **20**, 211.

HERMAN, T.S. (1983). Temperature dependence of adriamycin, *cis*-diamminedichloroplatinum, bleomycin, and 1,3-*bis*(2-chloroethyl)-1-nitrosourea cytotoxicity *in vitro*. *Cancer Res.*, **43**, 517.

HOLDENER, E.E., PARK, C.H., BELT, R.J., STEPHENS, R.L. & HOOGSTRATEN, B. (1982). Effect of mannitol and plasma on the cytotoxicity of cisplatin. *Eur. J. Cancer Clin. Oncol.*, 515.

LEROY, A.F., LUTZ, R.J., DEDRICK, R.L., LITTERST, C.L. & GUARINO, A.M. (1979). Pharmacokinetic study of *cis*-dichlorodiammine-platinum(II) in the beagle dog: Thermodynamic and kinetic behaviour of DDP in a biological milieu. *Cancer Treat. Rep.*, **63**, 59.

LITTERST, C.L., BERTOLERO, F. & UOZUMI, J. (1986). The role of glutathione and metallothionein in the toxicity and subcellular binding of cisplatin. In *Biochemical Mechanisms of Platinum Antitumour Drugs*, McBrian, D.C.H. & SLATER, T.F. (eds) p.227. IRL Press: Oxford.

MEIJER, C., MULDER, N.H., HOSPERS, G.A.P., UGES, D.R.A & DE VRIES, E.G.E. (1990). The role of glutathione in resistance to cisplatin in a human small cell lung cancer cell line. *Br. J. Cancer*, **62**, 72.

MELVIK, J.E., PETTERSEN, E.O., GORDON, P.B. & SEGLEN, P.O. (1986). Increase in *cis*-dichlorodiammineplatinum (II) cytotoxicity upon reversible electropermeabilization of the plasma membrane in cultured human NHIK 3025 cells. *Eur. J. Cancer Clin. Oncol.*, **22**, 1523.

MELVIK, J.E., DORNISH, J.M. & PETTERSEN, E.O. (1992). Characterization of a human cell line of cervix carcinoma origin, NHIK 3025/DPP, resistant to *cis*-diamminedichloroplatinum(II). *J. Cell. Pharmacol.*, **3**, 78.

MELVIK, J.E. & PETTERSEN, E.O. (1987). Reduction of *cis*-dichlorodiammineplatinum-induced cell inactivation by methionine. *Inorganica Chimica Acta*, **137**, 115.

MELVIK, J.E. & PETTERSEN, E.O. (1988). Oxygen- and temperature-dependent cytotoxic and radiosensitizing effects of *cis*-dichlorodiammineplatinum(II) on human NHIK 3025 cells *in vitro*. *Rad. Res.*, **114**, 489.

NEWMAN, A.D., RIDGWAY, H., SPEER, R.J. & HILL, J.M. (1979). Inhibition of biological activity of cisplatin by thiourea and L-methionine. *J. Clin. Hematol. Oncol.*, **9**, 208.

NORDBYE, K. & OFTEBRO, R. (1969). Establishment of four new cell strains from human uterine cervix. I. *Exp. Cell Res.*, **58**, 458.

OFTEBRO, R. & NORDBYE, K. (1969). Establishment of four new cell strains from human uterine cervix. II. *Exp. Cell Res.*, **58**, 459.

OVERGAARD, J. & KHAN, A.R. (1981). Selective enhancement of radiation response in a C3H mammary carcinoma by cisplatin. *Cancer Treat. Rep.*, **65**, 501.

PETTERSEN, E.O., BAKKE, O., LINDMO, T. & OFTEBRO, R. (1977). Cell cycle characteristics of synchronized and asynchronous populations of human cells and effect of cooling of selected mitotic cells. *Cell Tissue Kinet.*, **10**, 511.

PUCK, T.T., CIECIURA, S.J. & FISHER, H.W. (1957). Clonal growth *in vitro* of human cells with fibroblastic morphology. *J. Exp. Med.*, **106**, 145.

REPTA, A.J. & LONG, D.F. (1980). Reactions of cisplatin with human plasma and plasma fractions. In *Cisplatin: Current Status and New Developments*, Prestayko, A. W., Crooke, S.T. & Carter, S.K. (eds) p.285. Academic Press.

RIEMANN, F., ZIMMERMANN, U. & PILWAT, G. (1975). Release and uptake of haemoglobin and ions in red blood cells induced by dielectrical breakdown. *Biochim. Biophys. Acta*, **394**, 449.

ROBERTS, J.J. & FRAVAL, H.N.A. (1980). Repair of *cis*-platinum(II) diammine dichloride-induced DNA damage and cell sensitivity. In *Cisplatin: Current Status and New Developments*, Prestayko, A.W., Crooke, S.T. & Carter, S.K. (eds) p.57. Academic Press.

ROFSTAD, E.K., PETTERSEN, E.O., LINDMO, T. & OFTEBRO, R. (1980). The proliferation kinetics of NHIK 1922 cells *in vitro* and in solid tumours in athymic mice. *Cell Tissue Kinet.*, **13**, 163.

SEGAL, E. & LE PECQ, J.-B. (1985). Role of ligand exchange processes in the reaction kinetics of the antitumor drug *cis*-diamminedichloroplatinum(II) with its targets. *Cancer Res.*, **45**, 492.

SEKIYA, S., OOSAKI, T., ANDOH, S., SUZUKI, N., AKABOSHT, M. & TAKAMIZAWA, H. (1989). Mechanisms of resistance to *cis*-diammine-dichloroplatinum(II) in a rat ovarian carcinoma cell line. *Eur. J. Cancer Clin. Oncol.*, **25**, 429.

STRATFORD, I.J., WILLIAMSON, C. & ADAMS, G.E.(1980). Combination studies with misonidazole and a *cis*platinum complex: cytotoxicity and radiosensitization *in vitro*. *Br. J. Cancer*, **41**, 517.

SZUMIEL, I. & NIAS, A.H.W. (1976). Action of a platinum complex [cis-dichlorobis(cyclopentylamine)-platinum(II)] on Chinese hamster ovary cells *in vitro*. *Chem. Biol. Interact.*, **14**, 217.

TAKAHASHI, K., SEKI, T., NISHIKAWA, K. & 5 others (1985). Antitumor activity and toxicity of serum protein-bound platinum formed from cisplatin. *Jpn. J. Cancer Res.*, **76**, 68.

UCHIDA, K., TANAKA, Y., NISHIMURA, T., HASHIMOTO, Y., WATANABE, T. & HARADA, I. (1986). Effect of serum on inhibition of DNA synthesis in leukemia cells by *cis*- and *trans*-[Pt($NH_3$)$_2$$Cl_2$]. *Biochem. Biophys. Res. Comm.*, **138**, 631.

VAN DER VIJGH, W.J.F. & KLEIN, I. (1986). Protein binding of five platinum compounds. Comparison of two ultrafiltration systems. *Cancer Chemother. Pharmacol.*, **18**, 129.

ZIMMERMANN, U., VIENKEN, J. & PILWAT, G. (1980). Development of drug carrier systems: electrical field induced effects in cell membranes. *Bioelectrochem. & Bioenerget.*, **7**, 553.

ZIMMERMANN, U., SCHEURICH, P., PILWAT, G. & BENZ, R. (1981). Cells with manipulated functions: new perspectives for cell biology, medicine, and technology. *Angew. Chem. Int. Ed. Engl.*, **20**, 325.

ZWELLING, L.A. & KOHN, K.W. (1979). Mechanism of action of *cis*-dichlorodiammineplatinum(II). *Cancer Treat. Rep.*, **63**, 1439.